# NMI promotes hepatocellular carcinoma progression via BDKRB2 and MAPK/ERK pathway

**DOI:** 10.18632/oncotarget.14556

**Published:** 2017-01-06

**Authors:** Jing Zhao, Qiong-Zhu Dong, Fan Zhong, Li-Li Cai, Zhao-Yu Qin, Yang Liu, Cheng-Zhao Lin, Lun-Xiu Qin, Fu-Chu He

**Affiliations:** ^1^ Institutes of Biomedical Sciences, Fudan University, Shanghai, China; ^2^ Department of Systems Biology for Medicine, Shanghai Medical College, Fudan University, Shanghai, China; ^3^ Department of Surgery, Huashan Hospital, Cancer Metastasis Institute, Fudan University, Shanghai, China; ^4^ State Key Laboratory of Proteomics, Beijing Proteome Research Center, Beijing, China

**Keywords:** liver cancer, N-myc (and STAT) interactor (NMI), metastasis

## Abstract

Hepatocellular carcinoma (HCC) is one of the most prevalent and aggressive malignant tumors. The involvement of N-myc (and STAT) interactor (NMI) and its possible functional mechanisms in HCC progression still remain to be elucidated. In this study, we found that NMI was overexpressed in metastatic HCC cell lines compared with non-metastatic ones; and the expression levels of NMI in the HCC samples with metastasis were higher than that in the non-metastatic specimens. Furthermore, NMI depletion significantly decreased HCC cell proliferation and invasiveness *in vitro*, and also inhibited tumor growth and lung metastasis *in vivo* in nude mice models bearing human HCC. By contrast, NMI stable overexpression can enhance the malignant behaviors obviously. Moreover, we further verified that NMI promotes the expression of BDKRB2 and mediates the activation of MAPK/ERK signaling pathway according to the bidirectional perturbations of NMI expression *in vivo* or *in vitro* of HCC. Taken together, NMI is a pro-metastatic molecule and partially responsible for HCC tumor growth and motility. NMI could improve its downstream target BDKRB2 expression to induce ERK1/2 activation, and thereby further evoke malignant progression of HCC.

## INTRODUCTION

Hepatocellular carcinoma (HCC) is one of the most prevalent and aggressive malignant tumors worldwide [1]. Although survival of patients with HCC has been improved by surgical techniques and perioperative management, the prognosis of patients still remains very dismal mainly due to the high rate of recurrence and metastasis [2, 3]. The molecular pathogenesis and complicated signal transduction pathways implicated in HCC progression and metastasis are not fully understood, with currently only a few effectual diagnostic biomarkers and therapeutic targets [4].

Screening of independent biomarkers or pathways of metastatic HCC by using omics studies have become more popular due to technological advances in cell function monitoring from a holistic perspective [5]. Earlier studies focused on either transcripts or proteins, some of them reported marker genes or several superior predictors for HCC metastasis [6, 7], and other studies found the Akt/NFκB pathway and the HSP-centered network links to HCC [8, 9]. To better understand the metastatic drivers triggering HCC dissemination, we previously carried out an integrated transcriptomic and proteomic inspect among metastatic HCC cell lines, and identified 7 up-regulated significant genes including *NMI* [10].

N-myc (and STAT) interactor (NMI) is a protein which is encoded by *NMI* gene in human. It was first reported in 1996 that NMI interacts with *N-myc* and *c-Myc* oncogenes, and other transcription factors containing a Zip, HLH or HLH-Zip motif [11]. And then it was found to also can interact with all STATs except for STAT2 [12]. The roles of NMI in tumorigenesis, progress and metastasis are still in confusion. The expression of NMI has been inspected in 8 types of cancer cell lines. The expression of NMI in solid tumors was lower than that in myeloid leukemia and pancreatic ductal adenocarcinomas [11, 13]. A recent study showed that high expression of NMI predicted poor prognosis and promoted tumor growth in glioblastoma [14]. By contrast, it had also reported that NMI inhibited Wnt/β-catenin signaling pathway by up-regulating the expression of Dkk1, and then to block breast tumor growth. Loss of NMI promotes epithelial-mesenchymal transition of breast cancer [15–17]. The possible reason for the dual effects of NMI is presently unclear; it might in part due to the different tumor backgrounds of the patients. However, the function of NMI in HCC has never been reported.

In this study, we investigated the expression and function of NMI in HCC. We found that up-regulation of NMI was significantly associated with tumor metastasis in HCC. Moreover, *in vitro* and *in vivo* assays showed that NMI significantly promoted tumor proliferation, invasion, and metastasis of HCC by inducing its downstream target BDKRB2 expression and activator of MAPK/ERK signaling pathway. These results provide a clearer understanding of the underlying mechanism by which NMI promotes HCC metastasis and therapeutic target for HCC.

## RESULTS

### NMI expression level is associated with the metastatic potential of HCC

**Figure 1:**
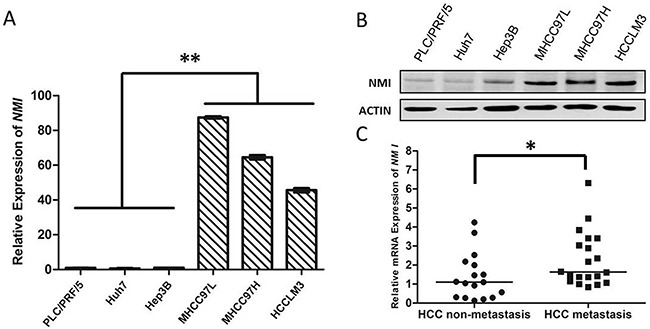
*NMI* expression is positively associated with HCC metastasis **A, B**. *NMI* mRNA expression and protein expression were detected in metastatic (MHCC-97L, MHCC-97H and HCC-LM3) and non-metastatic (PLC/PRF/5, Huh7 and Hep3B) HCC cell lines. **C**. In 37 HCC tumors specimens, the NMI expression of metastatic group (*n* = 20) was higher (*P < 0.05) than those of non-metastatic group (*n* = 17).

To evaluate the association of NMI with HCC metastasis, we analyzed NMI expression levels in a panel of human HCC cell lines with different metastatic potentials. Both the mRNA and protein levels of NMI in metastatic HCC cell lines (HCC-LM3, MHCC-97H, MHCC-97L) were much higher than those of the three non-metastatic HCC cell lines (PLC/PRF/5, Huh7, and Hep3B) by real-time PCR (Figure 1A) and Western blot (Figure 1B). These indicate that the overexpression of *NMI* correlates positively with metastatic potential of HCC cells.

To further evaluation the relationship between *NMI* expression and metastatic potential of HCC, we analyzed the mRNA expression of *NMI* in 20 metastatic primary HCC tissues and 17 non-metastatic HCCs by qRT-PCR (Table 1). Mann-Whitney U tests showed that the expression level of *NMI* in metastatic HCCs was apparently higher than that in those non-metastatic HCC tissues (*P < 0.05; Figure 1C).

**Table 1:** Clinicopathological correlations of NMI mRNA expression in HCC (*n* = 37)

Clinicpathological variables	# of HCC (*n* = 37)	*NMI* overexpression	*P* value
Gender			0.982
Male	30	13 (43.3%)	
Female	7	3 (42.9%)	
Age at diagnosis			0.199
>50	25	9 (36.0%)	
≤50	12	7 (58.3%)	
Tumor capsule			0.893
Positive	25	11 (44.0%)	
Negative	12	5 (41.7%)	
Tumor thrombi			0.603
Positive	18	7 (38.9%)	
Negative	19	9 (47.4%)	
Tumor size (cm)^1^			0.490
>5	28	13 (46.4%)	
≤5	9	3 (33.3%)	
Number of tumors			0.843
1	35	15 (42.9%)	
≥2	2	1 (50.0%)	
Tumor metastasis			0.004
Yes	20	13 (65.0%)	
No	17	3 (17.6%)	
AFP			0.338
>400	13	7 (53.8%)	
≤400	24	9 (37.5%)	

^1^Tumor size was represented by the length of the largest tumor nodule.

### NMI promotes *in vitro* proliferation, migration and invasion of HCC cells

**Figure 2:**
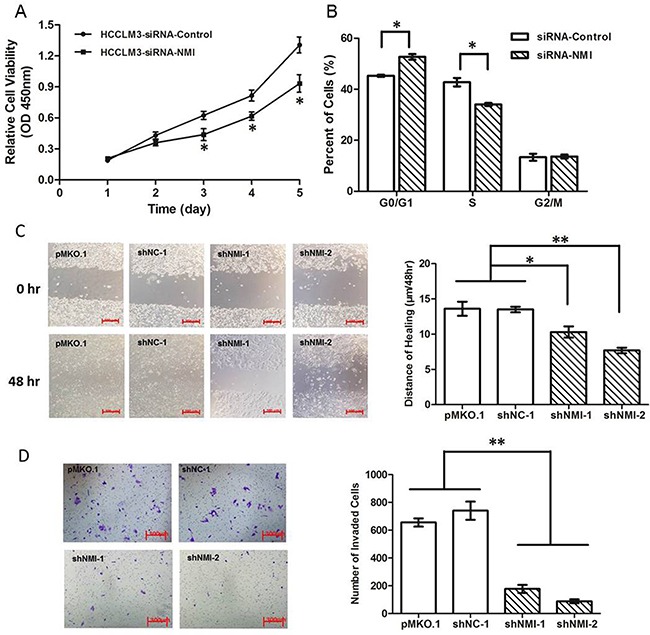
Knock down of NMI inhibited *in vitro* cell processes **A**. Cell growth rates in HCC-LM3 cells transfected with NMI siRNA were much less than that of those controls (*P < 0.05). **B**. Cell cycle G1/S arrest in HCC-LM3 cells transfected with NMI siRNA compared with controls (*P < 0.05). **C**. The distances of wound healing in NMI knock-down HCC-LM3 cells were shorter than of those controls in 48 hours (*P < 0.05). **D**. The *in vitro* invaded cell numbers were analyzed in HCC-LM3 cells transfected with shNMI or scrambled shRNA or empty vector (**P < 0.01).

**Figure 3:**
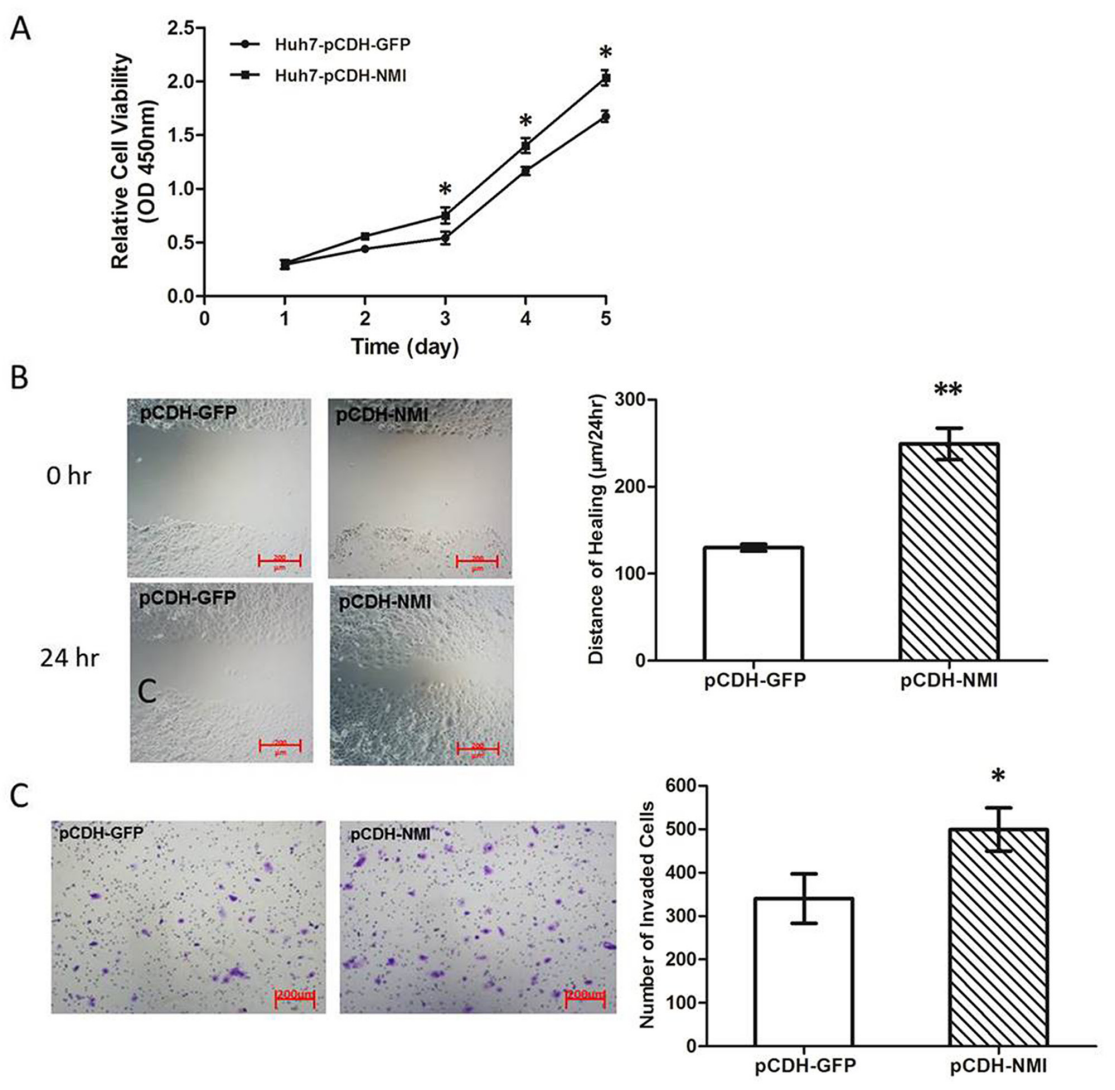
NMI overexpression promoted HCC cell progresses *in vitro* **A**. Cell growth rates in Huh7 cells with up-regulation of NMI were much larger than that of those controls (*P < 0.05). **B**. The distance of wound healing in Huh7 cells with up-regulation of NMI was longer than of those controls in 48 hours (**P < 0.01). **C**. The *in vitro* invaded cell numbers were analyzed in Huh7 cells transfected with shNMI or scrambled shRNA (*P < 0.05).

To explore the biological significance of NMI in HCC, we transfected an NMI expression plasmid or an anti-NMI siRNA vector into human HCC cell lines that have different endogenous NMI levels. Expression of NMI was verified by qRT-PCR and Western blotting (Supplementary Figure 1). Knock-down of NMI by siRNA in HCC-LM3 cell induced a significant suppression of cell proliferation (*P < 0.05; Figure 2A), an increase in percentage of G0/G1 phase and a decreased the percentage of S phase compared with the control group (*P < 0.05; Figure 2B); but did not have significant effect on the apoptosis of HCC-LM3 cells (Supplementary Figure 2). Moreover, NMI knock-down in HCC-LM3 cells also resulted in a significant delay in the wound closure rate detected by Scratch wound healing assay (*P < 0.05, **P < 0.01; Figure 2C), and a marked inhibition on the invasive ability of HCC cells in Matrigel invasion chamber assay (**P < 0.01; Figure 2D). These results demonstrate that *NMI* silencing is able to induce a G0/G1 arrest, significantly inhibit the abilities of *in vitro* cell proliferation, migration and invasion of HCC cells. In contrast, after up-regulation of NMI level by stably transfecting NMI expression vector into Huh7 cell line which has no metastatic potential and a lower endogenous expression level of NMI (Huh7-pCDH-NMI), the abilities of *in vitro* cell proliferation (Figure 3A), migration (**P < 0.01; Figure 3B), and invasion (*P < 0.05; Figure 3C) of Huh7-pCDH-NMI were markedly increased compare with the control Huh7-pCDH-GFP cells. Taking together, these indicate that *NMI* plays an important promoting role in the *in vitro* cell proliferation, migration and invasion of HCC cells.

### Effects of NMI on *in vivo* tumor growth and metastasis of HCC

**Figure 4:**
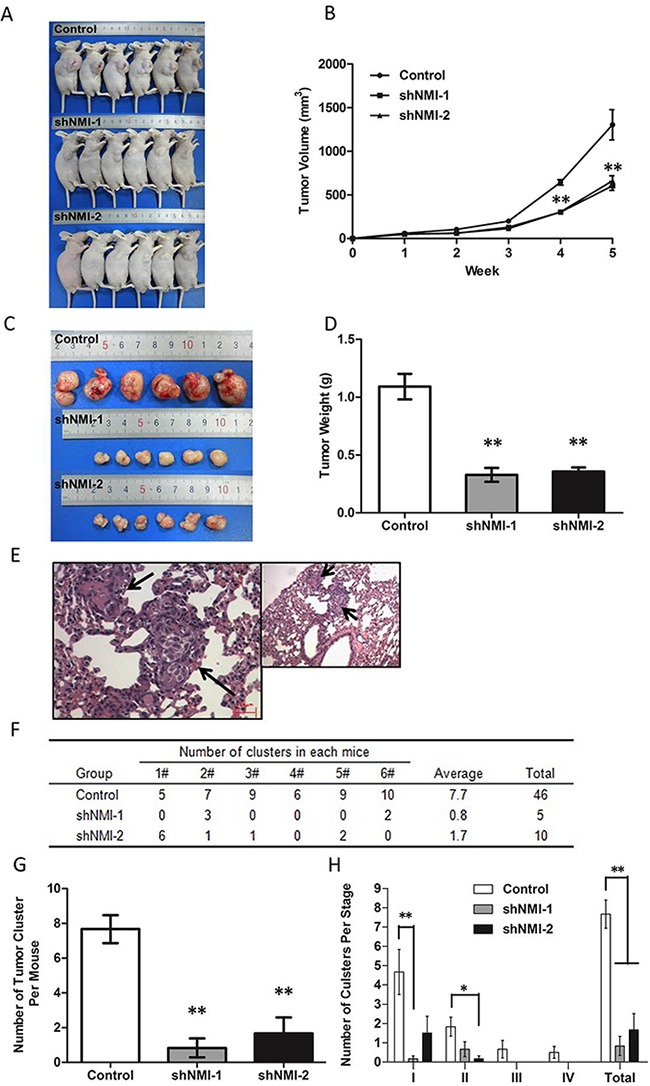
The effects of NMI on *in vivo* tumor growth and metastasis of HCC in a xenograft nude mice model **A-D**. Knockdown of NMI reduced the tumorigenicity. **A, C**. The tumor volumes derived from HCC-LM3 cell line stably transfected with shNMI or scrambled shRNA was measured *in vivo* for 5 weeks. (B) Both *in vivo* tumor growth rates and tumor size in mice models subcutaneously implanted with NMI knock-down HCC-LM3 cells were much slower than that of the controls after 3 weeks (**P < 0.01). (D) Weights of the xenograft tumors in models implanted with HCC-LM3 cells transfected with shNMI were much less than that of the controls (**P < 0.01). **E-H**. *NMI* silence decreased tumor metastasis *in vivo*. Effect of *NMI* on spontaneous metastasis was assayed via orthotopic implantation. (E) H&E staining of metastatic nodules formed at lung after *NMI* knockdown tumor tissue implantation into the liver with magnification of the selected areas. (Arrows: metastatic HCC nodules in the lung tissues). (F) All mice in control group had lung metastasis (100%, 6/6). The mice of the two lower *NMI* expression groups had less metastasis (33.3%, 2/6 and 66.7%, 4/6). (G) The number of tumor cluster of each experiment group mouse was significantly lower than control (**P < 0.01). (H) Staging of lung metastasis in HCC, the experimental group metastatic phases according to the cell number of metastatic lesion were significantly earlier than control group (*P < 0.05, **P < 0.01).

To evaluate the roles of NMI on *in vivo* tumor growth of HCC, we established a subcutaneous xenograft nude mice model using human HCC cells. After transfection of HCC-LM3 cell with shNMI-1 or shNMI-2, tumor size in mice models subcutaneously implanted with NMI-knockdown HCC-LM3 cells were much smaller than that of the controls after 3 weeks (**P < 0.01, Figure 4A-4B). The xenograft tumors were stripped and measured at the fifth week (Figure 4C). The average weights of the xenograft tumors in models implanted with HCC-LM3 cells transfected with shNMI (shNMI-1 0.328g±0.060g; shNMI-2 0.357g±0.036g) were much less than that of the controls (1.092g±0.111g) (**P < 0.01, Figure 4D). In the other word, knockdown of NMI induces a significant inhibition on the *in vivo* tumor growth of HCC-LM3 cells.

To further determine the effects of NMI on lung metastasis, the subcutaneous tumor xenografts were isolated and implanted into the liver to establish orthotopic models. Metastatic tumor nodules formed in the lungs were examined by H&E staining (Figure 4E). The incidences of lung metastasis in the shNMI-1 (33.3%, 2/6) and shNMI-2 (50%, 3/6) groups were significantly decreased compared with the control group (100%, 6/6) (**P < 0.01; Figure 4F). The number of tumor cluster of each experiment group mouse was significantly lower than control (**P < 0.01; Figure 4G). Moreover, based on the conventional grading and staging methods for lung metastasis of HCC nodules [18], in comparison to the controls in which all stages of lung metastases were able to found, only significantly earlier stages and decreased grades of metastatic lesions could be found in the two NMI knockdown groups (*P < 0.05, **P < 0.01; Figure 4H). Taking together, these suggest that *NMI* gene silence is able to significantly reduce the pulmonary metastatic ability of HCC-LM3 cells.

### NMI promotes the expression of BDKRB2

**Figure 5:**
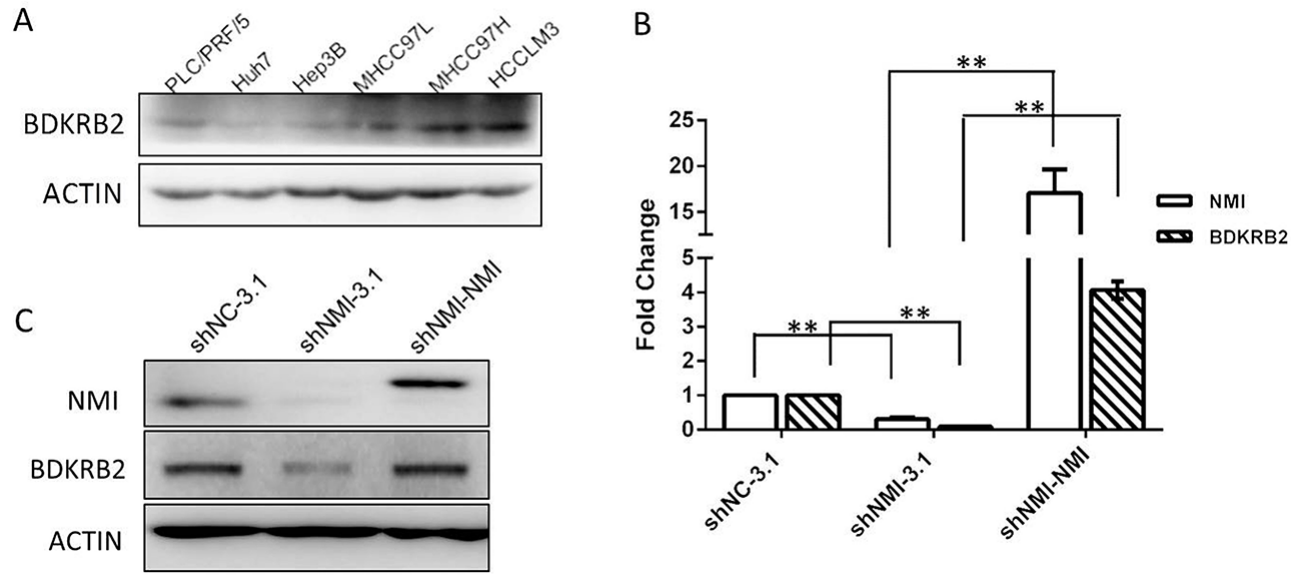
BDKRB2 may be the downstream target of NMI **A**. BDKRB2 protein expression were detected in metastatic (MHCC-97L, MHCC-97H and HCC-LM3) and non-metastatic (PLC/PRF/5, Huh7 and Hep3B) HCC cell lines. **B, C**. qRT-PCR and Western blotting showed the expression of BDKRB2 was significantly lower in NMI-knockdown HCC-LM3 cells than that of controls, and recovered the levels in the NMI-knockdown cells with re-transfected pcDNA3.1-*NMI* plasmid. NMI and BDKRB2 are positively correlated in HCC-LM3 cells.

To further investigate the role of NMI in HCC metastasis, the gene-expression profiles of NMI-knockdown HCC-LM3 were analyzed with Affymetrix Human Genome U133 Plus 2.0 Array. According to the criteria that t-test *P* < 0.05 and Fold Change > 2, significant alterations of expression levels were found in 1,205 genes after the knock-down of NMI in HCC-LM3 cells. Twenty two of them were further validated by qRT-PCR (Supplementary Figure 3). Furthermore, we analyzed these significant genes by Ingenuity Pathways Analysis (IPA, Ingenuity Systems Inc., USA) and found that Bradykinin receptor B2 is the most significantly changed after NMI down-regulation (FC = -98.819) (Supplementary Table 1-2). Bradykinin receptor B2, which is encoded by the BDKRB2 gene, is a G-protein coupled receptor for bradykinin in human as a specific cell surface membrane bradykinin type 2 constitutive receptor [19]. Thus, we found that BDKRB2 expression levels in the metastatic HCC cell lines were much higher than that in the non-metastatic ones (Figure 5A), and detected that the BDKRB2 transcript expression level was significantly down-regulated after NMI knockdown in HCC-LM3 by qRT-PCR (Supplementary Figure 4). BDKRB2 expression was also significantly decreased in the xenograft tumor tissues of shNMI groups tumors compared with that of control (Supplementary Figure 5). In addition, we transfected NMI plasmid to restore NMI level in the NMI-knockdown HCC-LM3 cells, and the high expression both mRNA and protein levels of BDKRB2 were significantly increased (Figure 5B–5C). These results indicate that BDKRB2 may be the downstream target of NMI.

### NMI activates MAPK/ERK signaling pathway

**Figure 6:**
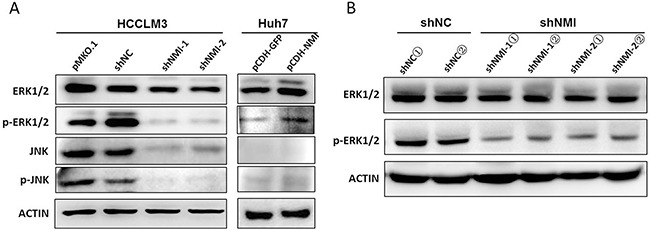
NMI induced MAPK/ERK signaling pathway **A**. Western blotting showed the ERK1/2 and phospho-ERK1/2 expressions in HCC-LM3 cells transfected with shNMI or Huh7 cells with up-regulation of NMI and their corresponding controls respectively. **B**. The expressions of phosphorylated EKR1/2 were decreased in subcutaneous tumors of mice models implanted with NMI knockdown HCC-LM3 cells compared with those of controls.

Next, we analyzed the signal pathway related to NMI function using Ingenuity Pathways Analysis, and found that most genes regulated by *NMI* are the downstream of MAPK/ERK pathway (Supplementary Figure 6). After NMI knockdown, the expression levels of MAPK signaling pathway members, including Raf1, MAPK11, MAP3K14 and MAP3K2, which are associated with cancer invasion and metastasis [20–22], were obviously decreased HCC-LM3 (Supplementary Table 3, Supplementary Figure 3). Given that BDKRB2 is widely known to be a G-protein coupled receptor of Bradykinin and is required in Bradykinin promoting the activation of MAPK/ERK pathway [23, 24]. We also evaluated the phosphorylation levels of three down signaling of MAPK pathway members (Supplementary Figure 7). We found that only ERK1/2 phosphorylation level obviously increased in Huh7 cells after up-regulation of NMI; and in contrast, significantly decreased phosphorylation levels of ERK1/2 in stable NMI silencing HCC-LM3 cells as well as their corresponding subcutaneous tumor tissues (Figure 6A, 6B). To identify whether BDKRB2 is involved in NMI-induced activation of MAP kinase signaling pathway, we assessed MAPK/ERK signaling pathway in NMI-upregulation HCC cells transfected with siBDKRB2 or scrambled siRNA and found that the reduced level of BDKRB2 led to the inactivation of ERK pathway (Supplementary Figure 8). The result demonstrated that BDKRB2 is a mediator for NMI-induced activation of MAPK/ERK signaling pathway.

## DISCUSSION

It has been demonstrated that NMI is widely expressed in fetal and adult tissues, and is overexpressed in multiple cell lines derived from hematolymphoid, hepatic and renal malignant tumors [11]. However, there is still controversial with its functional roles in cancer. High level of NMI expression was found in hematological malignant tumors, but it has relatively low expression in colon cancer, lung cancer and melanoma cell lines [12]. NMI is also reported to inhibit tumor growth through up-regulating Dkk1 [15], and loss of NMI promotes EMT by activation of TGFβ/SMAD signaling in breast cancer [16, 17]. Li et al. reported that NMI is a vital component of a transcription factor complex which allowed sustained activation of telomerase in breast and ovarian cancers [25]. NMI is a downstream target of the proto-oncogene Ets-1 which predicts poorer prognosis in breast, ovary and cervix cancers by regulated the expression of remodeling factors promoting invasive phenotype and disease progression [26, 27].

In our previous study, NMI was identified as one of the 7 leading up-regulated genes that were related to HCC metastasis [10]. In the present study, through investigating the expression levels of NMI in HCC cell lines with different metastatic potential and HCC tissues, we further confirm that up-regulation of NMI is significantly associated with HCC metastasis. But, the functional role of NMI in HCC is not clear yet. A recent study showed that NMI is a regulator of tumor proliferation which effects on glioma cell growth [14]. In the present study, through both the knock-down and restoration/transfection of *NMI* expression assays, we got a similar result that NMI is as a promotor of HCC growth by regulating the G0/G1 phase of cell cycle. Moreover, we found that NMI not only affected the tumor cell cycle progression, but also promoted tumor growth, invasion and metastasis through both *in vitro* and *in vivo* experiments. In summary, these results have clearly elucidated that NMI could be a pro-metastatic gene and is partially responsible for tumor growth and metastasis in HCC.

To further explore the possible mechanism of NMI in promoting HCC growth and metastasis, we performed gene-expression profiling analysis, and found that BDKRB2 was the most significantly changed gene regulated by NMI. Bradykinin is an inflammatory mediator and has recently been shown to mediate tumor growth and metastasis. A growing number of studies had investigated that BDKRB2 played a crucial role in bradykinin-regulating tumor progression [28, 29]. Bradykinin receptor B2 encoded by the *BDKRB2* gene is a G-protein coupled receptor for bradykinin in human. BDKRB2, as a specific cell surface membrane bradykinin type 2 constitutive receptor, is widely known to be a G-protein coupled receptor of Bradykinin which is required in Bradykinin promoting the activation of ERK/MAPK pathway. Using an antagonist of B2 receptor HOE-140, bradykinin could block the speed of glioma cell migration [30]. Repression of BDKRB2, but not B1 receptor, attenuated the bradykinin-mediated invasion and migration in colorectal cancer cells, and inhibited ERK1/2 activation and IL-6 production [31]. We observed that BDKRB2 expression was significantly decreased when NMI was knock-down in HCC-LM3 cells and its expression levels in the metastatic HCC cell lines were much higher than that in the non-metastatic ones. These suggest that BDKRB2 may be a target of NMI.

We found that many of significantly altered genes in *NMI* knockdown HCC-LM3 cells are regulated by ERK1/2 signaling. And most of these genes are reported to associate with cell adhesion, movement and angiogenesis. For instance, EGR1 (early growth response protein 1) regulates the activation of target genes as a nuclear transcription factor involving in cell differentiation and mitosis [32]. VCAM-1 (vascular cell adhesion molecule 1) promotes tumor angiogenesis [33]; and its mRNA expression was positively related to the lymph node metastasis. END 1 (Endothelin 1) can induce tumor protease activation and enhance migration and invasion of OVCA433 cells [34]. Metastasis suppressor gene *KiSS-1* is found a 2.5 folds increased expression in *NMI* knock-down HCC-LM3 cells. Moreover, NMI induced phosphorylation of ERK1/2 according to the bidirectional perturbations of NMI expression of HCC. Thus these evidences indicate that NMI induces ERK activation by promoting BDKRB2 expression; thereby further evoking HCC malignant progression.

In conclusion, our results show that NMI is a novel promotor of tumor growth, invasion and metastasis of HCC by inducing its downstream target BDKRB2 expression and activating MAPK/ERK signaling pathway. NMI may serve as a potential therapeutic target for combating HCC metastasis.

## MATERIALS AND METHODS

### Cell lines

The 3 HCC cell lines, MHCC-97L, MHCC-97H and HCC-LM3 with the same genetic background and stepwise increasing metastatic potentials were derived from the same patient. The 3 cell lines constitute a cellular metastasis model of human HCC [6, 18, 35]. For comparisons, we have also used another ordinary set of HCC cell lines, Hep 3B, PLC/PRF/5 and Huh7 [36–38], from American Type Culture Collection and Institute of Biochemistry and Cell Biology, Chinese Academy of Sciences, respectively. These cell lines routinely grew in DMEM High Glucose or RPMI 1640 (HyClone, Utah, USA) supplemented with 10% FBS in a 5% CO_2_ incubator at 37°C.

### Human HCC specimens

Human HCC tissues were obtained with informed consent from 37 patients who underwent curative liver resection for primary HCCs at authors’ institutes. Of the 37 cases with HCC, 20 had intrahepatic recurrence or distant metastasis within 3 years, and the other manifested no metastasis or recurrence. The primary HCC lesions which were accompanied with intrahepatic spreading, or tumor thrombosis in portal vein or bile duct were named as “metastatic HCC”. The diagnosis and histopathological features were confirmed by two pathologists independently. All specimens were frozen in liquid nitrogen immediately after resection and were stored at -80°C. The detail clinicopathologic characteristics of the patients are listed in Table 1. This study was approved by the Research Ethics Committee of Institutes of Biomedical Sciences, Fudan University (Shanghai, China).

### Animal models

Male athymic BALB/c nude mice (4~6 weeks, Shanghai Institute of Material Medicine, Chinese Academy of Science) were raised in specific pathogen-free conditions. Two different NMI silenced HCC-LM3 cell lines and their corresponding control cells were subcutaneously implanted into the subaxillary regions of left upper extremity of each mouse respectively.

For *in vivo* assays, subcutaneous tumor was removed and minced into small pieces with equal volumes (~1 mm^3^), and orthotopically transplanted into the livers of nude mice. All mice were monitored once every 3 days and sacrificed in 7 weeks. All experimental procedures involving animals were approved by The Animal Care and Use Committee of Fudan University, China.

### RNA isolation and qRT-PCR

Total RNA was isolated from cell lines and frozen tumor specimens by using Trizol reagent (Invitrogen). Cell cDNA was synthesized according to the protocol and proceeded to real-time PCR in the presence of SYBR Premix Ex Taq (TaKaRa) on the 7500 Real-Time PCR (Applied Biosystems). The primer sequences were as follows: sense 5’-TCCGGGAGTGCAGTCATCACG-3’ and anti-sense 5’-TCTCCACCTCCATTCTTTGCCCG-3’ for human *NMI*; sense 5’-CAGAGCCTCGCCTTTGCC-3’ and anti-sense 5’-ATGCCGGAGCCGTTGTCG-3’ for *ACTB*. Relative *NMI* mRNA expression levels were calculated based on the *Ct* values and normalized by *ACTB* expression level, according to the equation: 2^-Δ*Ct*^ [Δ*Ct* = *Ct* (*NMI*) - *Ct* (*ACTB*)]. *NMI* expression in HCC samples was measured by using TaqMan® Gene Expression Assays on ABI7900HT (Applied Biosystems). “Best Coverage” TaqMan® primer/probe set (Life Technologies) was ordered for *NMI*. We chose *HPRT1* as a reference gene, as it is the most reliable reference gene for q-PCR normalization in HBV-related HCC specimens [39]. All experiments had been done in triplicate.

### Establishment of cell lines with stable up- or down-regulation of NMI

To establish stable *NMI*-overexpressing or *NMI*-interfering cells, a full-length *NMI* or 2~3 shRNA sequences were cloned into pBABE or pCDH vectors. The recombinant vectors were cotransfected in HEK293T cells with VSVG and Gag using Lipofectamine 2000 (Life Technologies). Then the virus particles were harvested and used to infect Huh7 or HCC-LM3 cells. Stable pools were selected with puromycin (AMRESCO) for infected cells for 7 days. Virus vectors were used as controls. Stably transfected clones were validated by immunoblotting for NMI.

### SiRNA transient transfection

Three siRNAs against *NMI* were synthesized (Ribobio). The target sense sequences were: si-*NMI*-001: CCAAAGAAUUCCAGAUUAA; si-*NMI*-002: GAAAGU UCCUUAUGAGAUA; si-*NMI*-003: GAGUCAGAUU CCAGGUUUA. si-*NMI*s were transiently transfected into HCC-LM3 cells with Lipofectamine 2000 respectively, and the cellular lysates were prepared for Western blotting after transfection for more than 48 h.

### Western blotting analysis

Total cell lysates were generated from cell lines with RIPA buffer (50 mM Tris-HCl pH7.4, 150 mM NaCl, 1% Triton X-100, 1% sodium deoxycholate, 0.1% SDS) at the time of use to join 1X protease inhibitor cocktail (Roche). Protein concentrations were determined by BCA assay (Pierce). 40 μg of total cell lysates were loaded onto SDS-PAGE. Separated proteins were transferred onto nitrocellulose membranes (Millipore) and immunoblotted with appropriate antibodies. Proteins were detected using ECL detection system (Pierce). Anti-ACTIN (Santa Cruz) was used as the endogenous control. Primary antibodies used were as follows: NMI from Abgent; p-ERK1/2 (Thr202/Tyr204), ERK1/2, p-JNK (Thr183/Tyr185), JNK and BDKRB2 from Signaling Technology; HRP-conjugated anti-rabbit and anti-mouse secondary antibodies (Pierce).

### Cell proliferation, migration, and invasion assays

Cells were dispensed in 100-μL aliquots into a 96-well plate with about 2,500 cells/well. Each of these cells was inoculated 6 holes. At the indicated time points, 10μL CCK-8 solution (Dojindo, Japan) was added to each well of the plate, and further incubated for 2 h. The absorbance at 450 nm was measured by using a microplate reader. All experiments were triply repeated.

Cell migration was evaluated by using the scratch wound assay. Cells were cultured in 24-well plate for 1 or 2 days to form a tight cell monolayer, and then the cell monolayer was scratched with a 200 μL plastic pipette tip to produce a wound gap. The remaining cells were washed twice with culture medium to remove cell debris and incubated at 37°C with serum-free medium. At the indicated times, migrating cells at the scratches were photographed using an inverted microscope (Olympus). The percentage of the cleared area at each time point compared with time 0 was measured using Image-Pro Plus v6.0 (Media Cybernetics).

The invasive ability of HCC cells was determined using 24-well transwell chambers, 8 μm pore size, coated with thin Matrigel (BD Biosciences). The transfected cells were suspended in 100 μL serum-free DMEM and were added to the top of the gels in the triplicate chambers. The bottom chamber was filled with 600 μL DMEM with 10% FBS. After 48 hours of incubation, the Matrigel and the cells remaining in the upper chamber were removed by cotton swabs. Cells on the lower surface of the membrane were fixed in 4% paraformaldehyde and stained with Giemsa. Cells in five microscopic fields (at 200× magnification) were counted and photographed with an inverted microscope. All experiments were performed in triplicate.

### *In vivo* assays for tumor growth and metastasis

HCC-LM3-sh*NMI*-1, HCC-LM3-sh*NMI*-2 and HCC-LM3-Mock (~4×10^6^ cells) were suspended in 200 μL PBS and then injected subcutaneously into the upper left flank region of nude mice (*n* = 6). Tumor sizes were measured every week (volume = *ab*^2^/2, where *a* and *b* are the longest and the shortest perpendicular diameters of the tumor respectively), and tumor weights were taken at termination day. Tumor was removed and minced into small pieces with equal volumes (~1 mm^3^), and orthotopically transplanted into the livers of nude mice. All mice were monitored once every 3 days and sacrificed in 7 weeks. These procedures were approved by the Animal Care and Use Committee of Fudan University (China). Lungs were removed and embedded in paraffin and the total number of lung metastases was counted under the microscope as described previously [40]. And the metastases were classified into four grades basing on the number of tumor cells present at the maximal section of each metastatic lesion: grade I, ≤20 tumor cells; grade II, 20~50 tumor cells; grade III, 50~100 tumor cells; grade IV, >100 tumor cells [18, 40].

### Statistical analysis

Clinical HCC specimens were analyzed by Mann-Whitney U tests. All *in vitro* experiments were conducted in triplicate and carried out on three separate occasions. Statistically significant differences were determined by two-tailed unpaired t-test. *P* < 0.05 was considered statistically significant.

## SUPPLEMENTARY MATERIALS FIGURES AND TABLES


