# Supplementary material for: NMI promotes hepatocellular carcinoma progression via BDKRB2 and MAPK/ERK pathway
Source: Oncotarget. 2017 Jan 6;8(7):12174–85. doi: 10.18632/oncotarget.14556 (PMC5355334; doi:10.18632/oncotarget.14556)
Supplement: Supplementary file 1 [file oncotarget-08-12174-s001.pdf]

NMI promotes hepatocellular carcinoma progression via BDKRB2 and MAPK/ERK pathway

SUPPLEMENTARY FIGURES AND TABLES

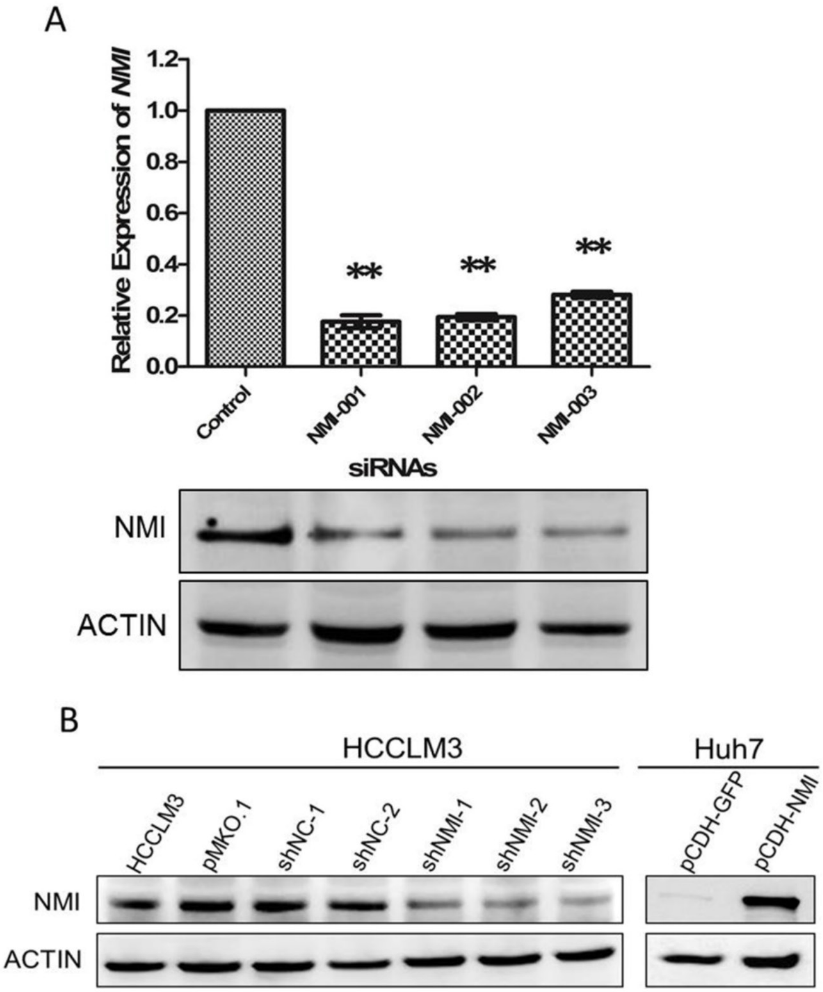

Supplementary Figure 1: qRT-PCR and Western blotting confirmed *NMI* expression in transiently and stably transfected and parent cells. A. Relative mRNA and protein expressions of *NMI* siRNAs or negative control siRNA transfected HCC-LM3 cells; **B.** The stable cell lines inhibiting NMI in HCC-LM3 and NMI overexpression in Huh7 were constructed through shRNA transfection.

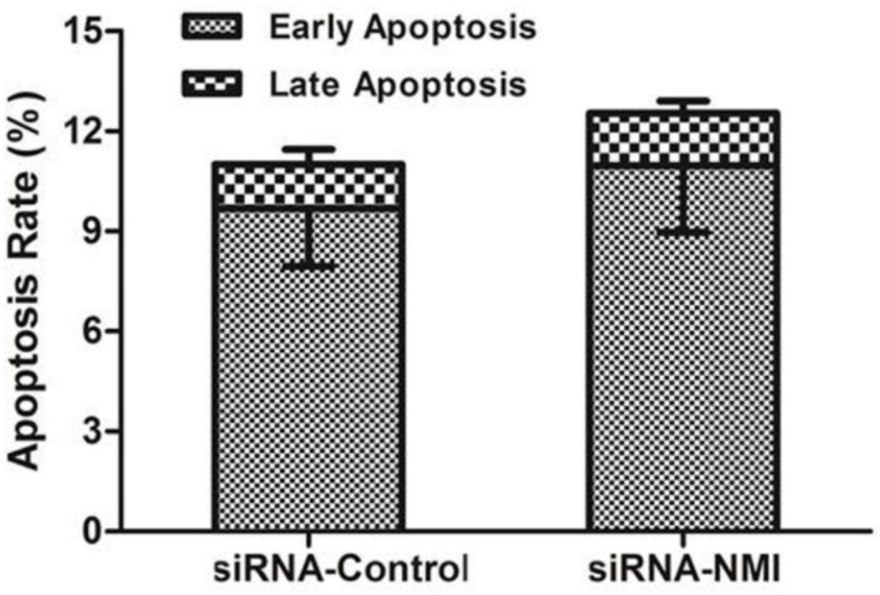

Supplementary Figure 2: Knock-down of NMI did not have any significant influence on apoptosis rate in HCC-LM3. The cell apoptosis assay was validated by flow cytometer.

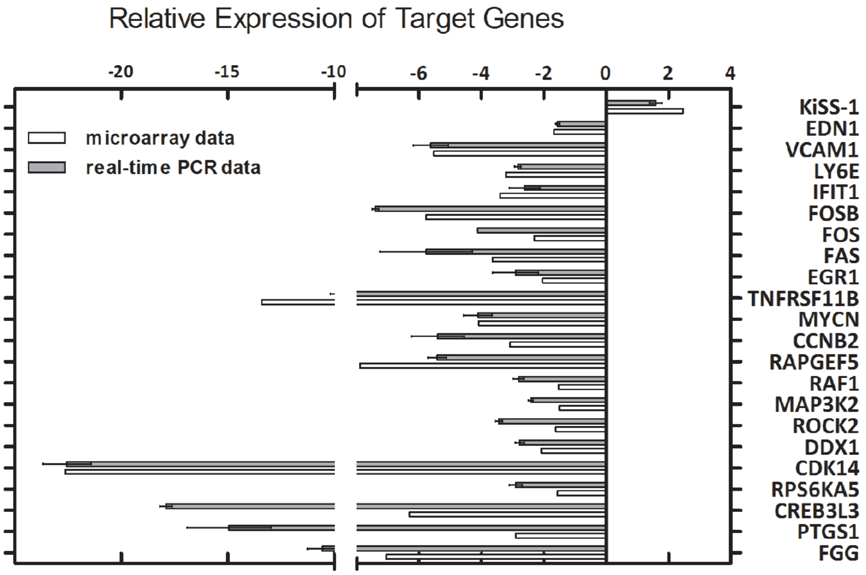

Supplementary Figure 3: qRT-PCR validated 22 significantly changed genes of stable *NMI*-knockdown HCC-LM3. Their changes in the expression of mRNA were consistent with the microarray results. Many genes were reported to associate with tumor cell proliferation, invasion and metastasis. The molecules involve cell adhesion molecules (ICAM), cell movement related molecules and angiogenesis related ones. Some genes were reported to be regulated by ERK1/2 signaling.

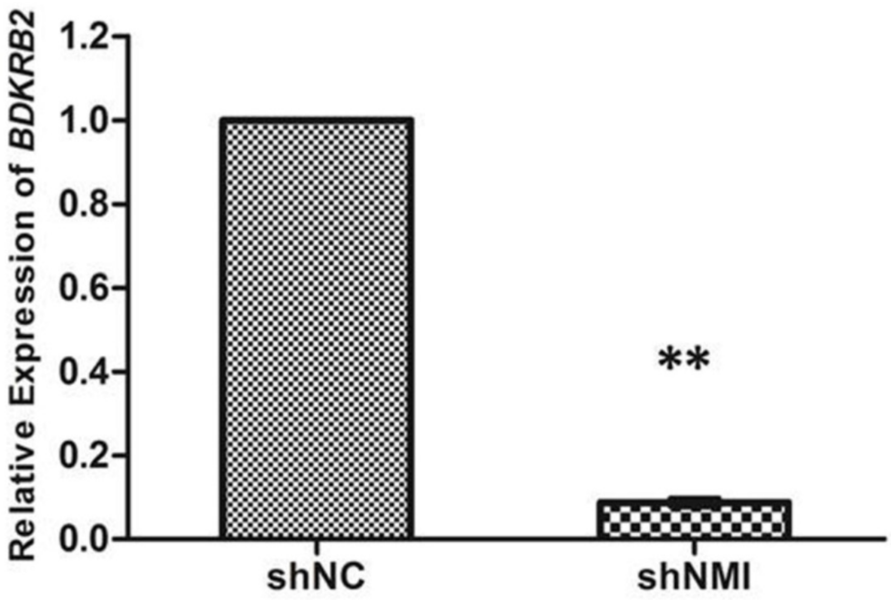

Supplementary Figure 4: qRT-PCR confirmed the BDKRB2 expression is significant decreased in NMI knockdown HCC-LM3.

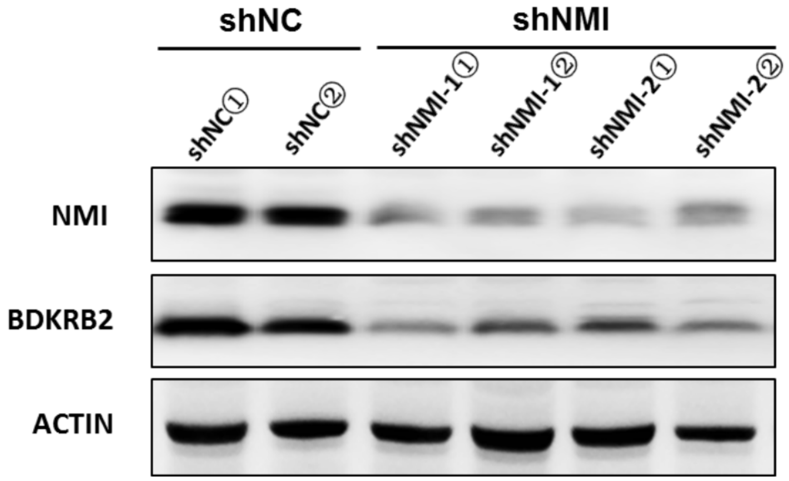

Supplementary Figure 5: Western blotting showed BDKRB2 expression in xenograft tumors in mice models implanted with HCC-LM3 cells transfected with shNMI and scrambled shRNA.

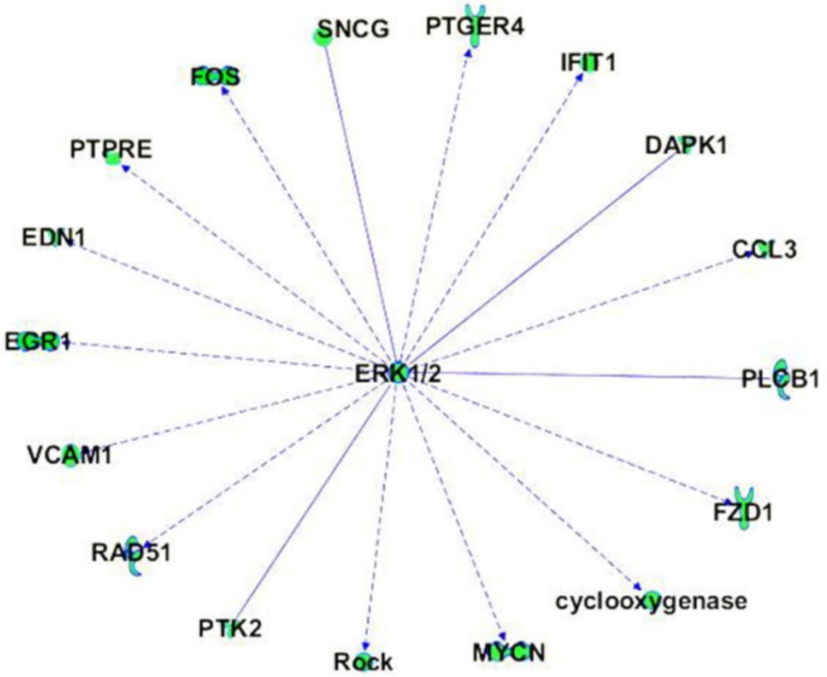

Supplementary Figure 6: From IPA network analysis, the expressions of most altered proteins in stable *NMI*-knockdown HCC-LM3 are regulated by ERK1/2.

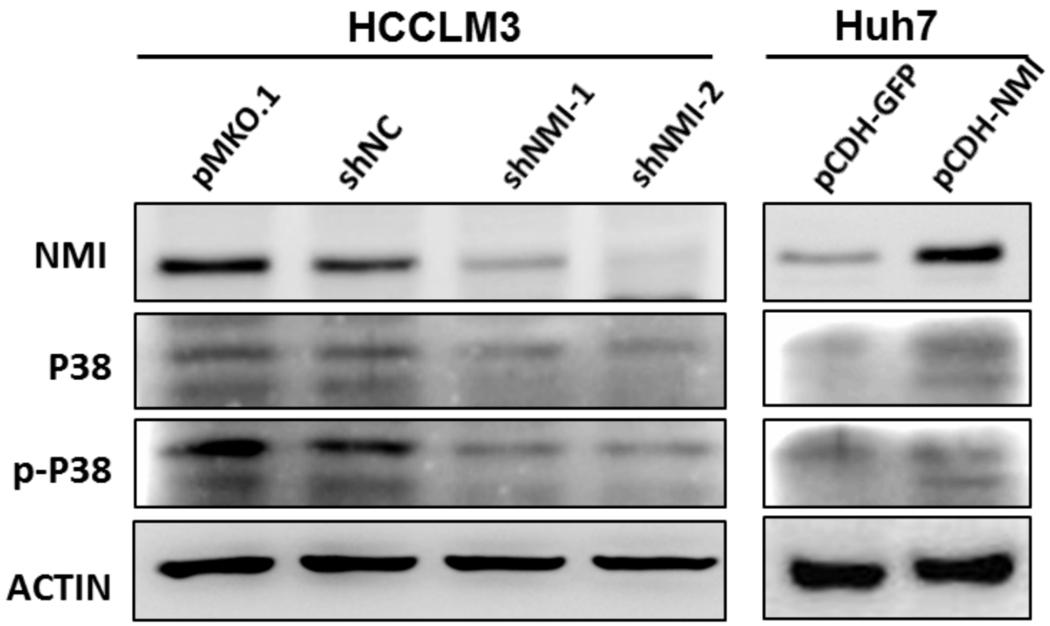

Supplementary Figure 7: Western blotting showed the expression of p38, phospho-p38 and NMI in HCC-LM3 cells transfected with shNMI or Huh7 cells with up-regulation of NMI and their corresponding controls respectively.

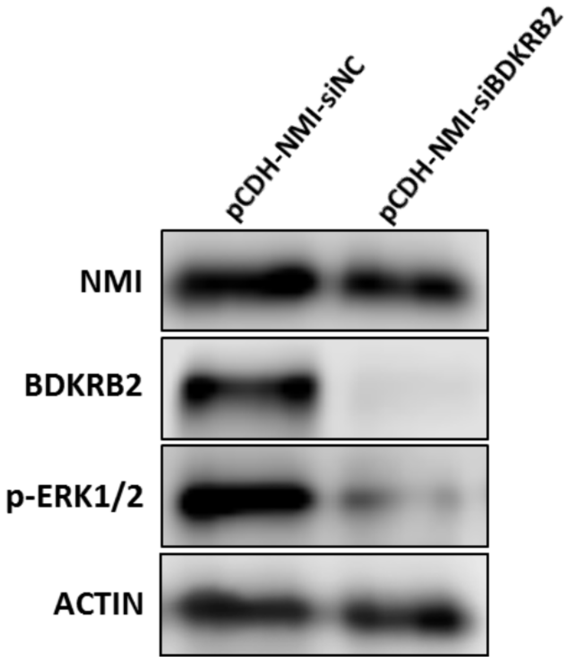

Supplementary Figure 8: Western blotting showed the expressions of BDKRB2 and its related molecules, NMI and phospho-ERK1/2, in the NMI-upregulation Huh7 cells transiently transfected BDKRB2 siRNA.

Supplementary Table 1: The top twenty genes changed significantly from transcriptome screening

Probe Name	Gene Symbol	Description	P Value	Fold Change
A_23_ P171074	ITM2A	Homo sapiens integral membrane protein 2A (ITM2A)	1.17E-05	0.001558017
A_24_ P153342			0.001174362	0.005066744
A_23_ P121926	SEPP1	Homo sapiens selenoprotein P, plasma, 1 (SEPP1)	0.001548515	0.00507055
A_24_ P276073	MGC10814	Homo sapiens hypothetical protein MGC10814, mRNA (cDNA clone MGC:10814 IMAGE:3613095)	0.006291837	0.00622659
A_24_ P920715			0.002589019	0.006377919
A_23_ P26854	ARHGAP44	Homo sapiens Rho GTPase activating protein 44 (ARHGAP44)	0.000408013	0.007048203
A_23_ P120902	LGALS2	Homo sapiens lectin, galactoside-binding, soluble, 2 (LGALS2)	1.60E-05	0.00726422
A_24_ P375911	CXorf18	Homo sapiens chromosome X open reading frame 18 (CXorf18), non-coding RNA	0.000271532	0.007628033
A_23_ P27040	TMEM98	Homo sapiens transmembrane protein 98 (TMEM98)	2.14E-06	0.007962394
A_23_ P27035	TMEM98	Homo sapiens transmembrane protein 98 (TMEM98)	0.002605303	0.008104393
A_23_ P251151	NELL1	Homo sapiens NEL-like 1 (chicken) (NELL1)	0.000534454	0.009762165
A_23_ P304897	BDKRB2	Homo sapiens bradykinin receptor B2 (BDKRB2)	0.023198496	0.010119509
A_23_ P313623	COBL	Homo sapiens cordon-bleu homolog (mouse) (COBL)	0.001302853	0.013005537
A_24_ P55295	GJA1	Homo sapiens gap junction protein, alpha 1, 43kDa (GJA1)	6.97E-05	0.014806161
A_23_ P253896	NPNT	Homo sapiens nephronectin (NPNT)	0.00028267	0.016865249
A_23_ P431923	SSX8	Homo sapiens synovial sarcoma, X breakpoint 8 (SSX8), non-coding RNA	0.001978126	124.7747639
A_32_ P55135	SSX1	Homo sapiens synovial sarcoma, X breakpoint 1 (SSX1)	0.001807352	95.89857947
A_23_ P114134	SSX3	Homo sapiens synovial sarcoma, X breakpoint 3 (SSX3)	0.009142994	92.02037452
A_32_ P101031	LYPD1	Homo sapiens LY6/PLAUR domain containing 1 (LYPD1)	0.000249314	75.10927211
A_23_ P33881	SSX4B	Homo sapiens synovial sarcoma, X breakpoint 4B (SSX4B)	0.001794072	70.86545266

Supplementary Table 2: 11 molecule(s) associated with coagulation system which is the most significant pathway in the canonical pathway identified by IPA

Probe Name	Gene Symbol	Description	P Value	Fold Change
A_23_ P304897	BDKRB2	Homo sapiens bradykinin receptor B2 (BDKRB2)	0.023198496	0.010119509
A_23_ P205355	SERPINA5	Homo sapiens serpin peptidase inhibitor, clade A (alpha-1 antiproteinase, antitrypsin)	0.005072552	0.039240251
A_23_ P218111	SERPINA1	Homo sapiens serpin peptidase inhibitor, clade A (alpha-1 antiproteinase, antitrypsin)	0.003789585	0.13735017
A_23_ P148088	FGG	Homo sapiens fibrinogen gamma chain (FGG)	0.000326108	0.141586218
A_23_ P375372	FGA	Homo sapiens fibrinogen alpha chain (FGA)	0.026023085	0.256678117
A_32_ P41604	F5	Homo sapiens coagulation factor V (proaccelerin, labile factor) (F5)	0.044987759	0.450796044
A_23_ P91390	THBD	Homo sapiens thrombomodulin (THBD)	0.004671107	0.472151194
A_23_ P24104	PLAU	Homo sapiens plasminogen activator, urokinase (PLAU)	0.01342242	2.85422415
A_24_ P383480	PROS1	Homo sapiens protein S (alpha) (PROS1)	8.35E-05	3.486285583
A_23_P6335	SERPIND1	Homo sapiens serpin peptidase inhibitor, clade D (heparin cofactor)	0.029297959	4.762020729
A_23_ P82868	PLAT	Homo sapiens plasminogen activator, tissue (PLAT)	0.013086817	6.914456925

Supplementary Table 3: The MAPK pathway gene shortlist from transcriptome screening

Probe Name	Gene Symbol	Description	P Value	Fold Change
A_23_ P40952	RAF1	Homo sapiens v-raf-1 murine leukemia viral oncogene homolog 1 (RAF1)	0.003289391	0.656494794
A_23_ P45025	MAPK10	Homo sapiens mitogen-activated protein kinase 10 (MAPK10)	0.028812123	0.659031109
A_23_ P502274	MAPK11	Homo sapiens mitogen-activated protein kinase 11 (MAPK11)	0.03615372	0.553848496
A_23_ P502078	MAPK8IP2	Homo sapiens mitogen-activated protein kinase 8 interacting protein 2 (MAPK8IP2)	0.028200489	0.591359298
A_24_ P272594	MAPKBP1	Homo sapiens mitogen-activated protein kinase binding protein 1 (MAPKBP1)	0.021975413	0.56309502
A_23_ P58031	MAP3K13	Homo sapiens mitogen-activated protein kinase kinase kinase 13 (MAP3K13)	0.024083475	0.520223017
A_23_ P207319	MAP3K14	Homo sapiens mitogen-activated protein kinase kinase kinase 14 (MAP3K14)	0.021472743	0.557292516
A_32_ P98887	MAP3K2	Homo sapiens mitogen-activated protein kinase kinase kinase 2 (MAP3K2)	0.011158954	0.666198597
